# Application of a high-resolution melting technique for the rapid detection of partial replacement of HCV-1b by HCV-1a after PEG-IFNα/RBV therapy

**DOI:** 10.1007/s13353-014-0256-3

**Published:** 2014-11-08

**Authors:** M. Holysz, K. Bialas, P. Migdalski, D. Kmieciak, W. H. Trzeciak

**Affiliations:** 1 Department of Biochemistry and Molecular Biology, Poznan University of Medical Sciences, Poznan, Poland; 2 Department of Infectious Disease, Poznan University of Medical Sciences, Poznan, Poland

**Keywords:** HCV-1b conversion, HCV-1a, High-resolution melting

## Abstract

A modified method which can be used for the rapid screening of mutations in the protein kinase R-binding domain (PKR-BD) region and the hypervariable region 1 (HVR1) of hepatitis C virus (HCV) is described. This method is based on a high-resolution melting (HRM) technique used for genotyping single nucleotide polymorphisms and allows the detection of single nucleotide substitutions in the DNA sequence by measuring its T_m_. The modified method, in addition to precisely measuring the T_m_, allows the recording of the melting curve of the investigated cDNA fragment, which can provide provisional information about the number of different quasi-species present in the sample. The HRM analysis of the amplified cDNAs encoding the PKR-BD and HVR1 allowed the detection of partial replacement of HCV-1b by HCV-1a subspecies in one of our patients, as well as evaluation of the effectiveness of pegylated interferon α/ribavirin (PEG-IFNα/RBV) therapy. The HRM technique has never been used for the rapid screening of sequence variations in these regions and may be used for a similar purpose in any viral genome.

## Introduction

Routine therapy of hepatitis C virus (HCV), based on pegylated interferon α combined with ribavirin (PEG-IFNα/RBV), enables the achievement of a sustained virologic response (SVR) in only about 50 % of patients suitable for this type of treatment (Fried et al. 2002). A three-drug therapy is presently used. The serine protease inhibitors telaprevir (TVR) or boceprevir (BEC) are currently added to standard PEG-IFNα/RBV treatment, resulting in the improvement of SVR to over 70 % (Kozielewicz et al. 2012).

Numerous genetic studies have demonstrated that the effects of treatment mostly depend on the genotype of the virus, as well as on its ability to create new strains during the course of infection (Holland et al. 1992; Noppornpanth et al. 2007). Therefore, rapid detection of new subspecies of the virus is of critical importance. Currently used methods involve reverse transcription of viral RNA, followed by amplification of cDNA by quantitative reverse transcription polymerase chain reaction (RT-qPCR). The amplified cDNA is then cloned, sequenced, and converted into an amino acid (aa.) sequence of viral protein. This method is laborious, time-consuming, and expensive, and there is a need for a method which would allow one to distinguish between the subspecies of the virus rapidly and at a low cost.

The high-resolution melting (HRM) technique, which has been successfully used for the genotyping of single nucleotide polymorphisms, allows one to detect even a single nucleotide substitution in the DNA sequence by recording the melting curve of the investigated DNA and measuring its T_m_.

Our report illustrates the importance of the HRM technique for the rapid detection of the conversion of HCV-1b into HCV-1a subspecies in a patient subjected to PEG-IFNα/RBV therapy.

## Materials and methods

### Patient and treatment

Our patient was selected from a group of about 40 patients who underwent unsuccessful standard therapy with PEG-IFNα-2a/RBV. Grading and staging levels as well as liver biopsy confirmed the diagnosis of chronic hepatitis, and infections with hepatitis B virus (HBV) or other hepatotropic viruses were excluded. The Local Ethical Committee approved the study and written consent was obtained from the patient. The study adheres to the principles of the Declaration of Helsinki.

### Reverse transcription and amplification of cDNA

Total RNA was extracted (Chomczynski and Sacchi 1987) from the sera (collected before and after 12 months of therapy) and reversely transcribed with specific primers 5′-TWGTRGCGAGCTCCGCCAAG-3′ [for the protein kinase R-binding domain (PKR-BD)] and 5′-GCCGAAACGGTCGGTCGT-3′ [for the hypervariable region 1 (HVR1)]. The cDNAs encoding the PKR-DB region and HVR1 were PCR-amplified with the following primer pairs: 5′-TCCTTGGCCAGCTCHTCAGC-3′ and 5′-TGGGCAWCGCTGGRGGGAA-3′ (for PKR-BD), and 5′-AGGTBYTGATTGTGATGCTAC-3′ and 5′-AGTCATTGCAGTTCAGGGC-3′ (for HVR1), at an optimal annealing temperature determined by using CFX96 (Bio-Rad, USA). Specific primers were designed according to the reference sequence D90208 (Kato et al. 1990; Kmieciak et al. 2006).

### Real-time PCR HRM analysis

HRM analysis was preceded by qPCR amplification of cDNA encoding PKR-BD and HVR1 in the LightCycler 480 II (Roche Diagnostics, Germany), using the LightCycler 480 High-Resolution Master Kit (Roche Diagnostics, Germany). The following protocol was adopted: preincubation 95 °C, 10 min and amplification 95 °C, 10 s; 60 °C, 20 s; 72 °C, 10 s (with a single fluorescence measurement), 50 cycles, with subsequent HRM analysis: 95 °C, 1 min; 40 °C, 1 min. The melting curve was recorded from 70 to 99 °C, increasing at a rate of 0.02 °C/s with continuous fluorescence measurement. The melting curves were analyzed with the Gene Scanning module (Roche Diagnostics, Germany).

### Cloning and sequencing

In order to amplify cDNAs encoding PKR-BD and HVR1, semi-nested PCR was used. The PCR products were purified and digested with the restriction endonucleases ClaI and XhoI for PKR-BD and HindIII and PstI for HVR1. The fragments were ligated into a pBluescript SK+/− vector and the resulting constructs were used to transform *Escherichia coli* cells (Strain XL-1 Blue). Recombinant clones were cultured in Luria-Bertani (LB) broth, and plasmids were isolated and sent for sequencing. The nucleotide sequences were converted into an aa. sequence with the program OMIGA 2.0.

## Results

HRM analysis was applied to screen for alterations in the nucleotide sequences of cDNAs encoding the PKR-BD region and the HVR1 in our patient. These two regions were chosen because the PKR-BD regions contains an interferon sensitivity-determining region (ISDR) and mutations in this region might play a crucial role in the resistance to PEG-IFNα/RBV therapy, whereas the HVR1 is highly variable and mutations in this region reflect the ineffectiveness of therapy (Kmieciak et al. 2006).

Alterations in the nucleotide sequences were visualized as changes in the melting curves and the T_m_ of the qPCR products, recorded at the beginning of the therapy and after 12 months of treatment. The results obtained by HRM analysis of the cDNAs were confirmed by direct sequencing.

### Analysis of cDNA fragment encoding the PKR-BD region

**Fig. 1:**
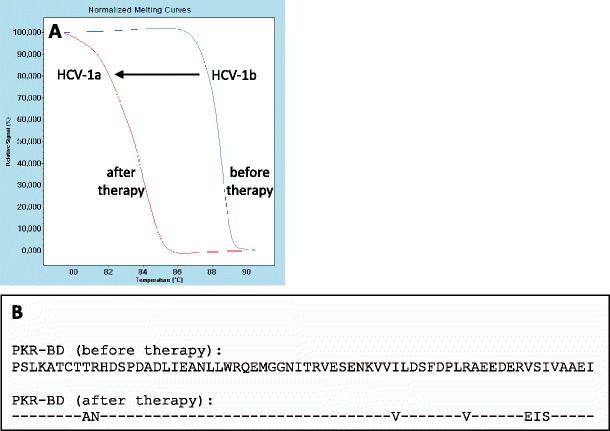
Analysis of cDNA encoding the protein kinase R-binding domain (PKR-BD) region before and after the pegylated interferon α/ribavirin (PEG-IFNα/RBV) therapy. **a** Melting curve before and after the therapy. The *arrow* indicates the T_m_ shift from before to after the therapy. **b** aa. sequence of the C-terminal region of the NS5A protein covering the PKR-BD region before and after the therapy

The melting curves of the qPCR products, covering the entire coding sequence of the C-terminal region of the NS5A protein obtained for pairs of samples taken before and after therapy from about 40 patients, revealed a single qPCR product, which melts at 88.5 °C, typical of the HCV-1b subtype. In our patient, the shape of the melting curve and the T_m_ before therapy were almost identical to those of other patients. However, after the therapy, we found a shift in the melting curve and a change in the T_m_ to 84 °C, which is typical of the HCV-1a variant (Fig. 1a). This suggested that the change from the 1b subtype before therapy to the 1a subtype after treatment had taken place.

The amplification product of the PKR-BD cDNA was cloned and sequenced, and the sequence was converted into an aa. sequence. In our patient, before therapy, there was no change in the aa. sequence of the PKR-BD region, which was typical of HCV-1b. After therapy, seven aa. substitutions were detected, resulting in the sequence characteristic for the HCV-1a subtype (Fig. 1b). This sequence shows 97 % homology (64 out of 66 aa. were identical) to the original one deposited in GenBank. We concluded that, in this patient, as a consequence of the therapy, the HCV-1b subtype was partially replaced by the HCV-1a subtype.

### Analysis of cDNA fragment encoding the HVR1

The HRM analysis, covering the HVR1, which encodes 27 N-terminal aa. of the envelope protein E2, revealed one major peak of irregular shape.

According to the sequence diversity of different HCV-1b quasi-species, the analysis of melting curves of HVR1 cDNA should show multiple amplification products identified as separate peaks of different T_m_. In our patient, the melting curve before therapy revealed a single major peak of irregular shape and a T_m_ of 89.8 °C. After therapy, the shape of the melting curve remained unchanged, but a shift in the T_m_ to 91 °C was observed, suggesting the appearance of a different species of the virus.

**Fig. 2:**
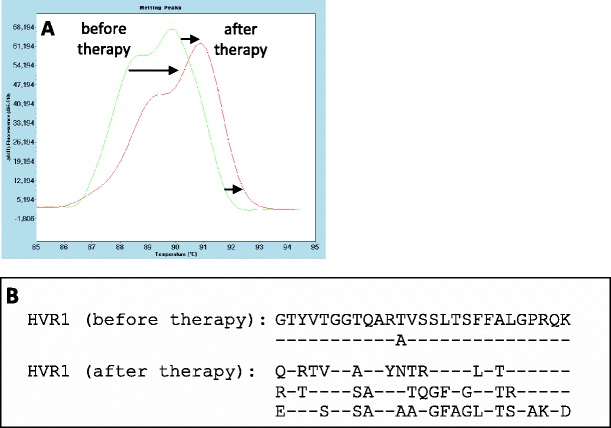
Analysis of cDNA encoding the hypervariable region 1 (HVR1) before and after the PEG-IFNα/RBV therapy. **a** Melting peaks before and after the therapy. The *arrows* indicate the T_m_ shift from before to after the therapy. **b** aa. sequence of the HVR1 before and after the therapy

Sequence analysis of HVR1 cDNA before therapy revealed two nearly identical (difference of 1 aa.) strains typical of HCV-1b. After therapy, two strains of 59 % homology and one of 40 % homology to the initial strains were detected. The last strain showed 85 % homology to the HCV-1a subtype (Fig. 2b), according to the aa. sequence deposited in GenBank, confirming that partial replacement of HCV-1b by HCV-1a had taken place.

## Discussion

The HRM analysis showed that, in our patient, the shape of the melting curves of the PKR-BD cDNA was different before and after treatment, and the T_m_ changed from 88.5 °C to 84 °C, respectively, indicating alterations in the nucleotide sequence of PKR-BD cDNA during the course of treatment.

In most cases, we have found that single transversions within complementary base pairs (e.g., AT↔TA or GC↔CG) usually affect the melting curve to a small extent (less than 0.2 °C), but multiple mutations, which is the rule in the case of HCV DNA, result in appreciable changes in the T_m_. Therefore, a difference of over 4 °C in T_m_ meant a major change in the nucleotide sequence.

Before the therapy, the aa. sequence of the PKR-BD region was typical of HCV-1b. After therapy, however, seven aa. substitutions were detected, resulting in a sequence characteristic of the HCV-1a subtype. This suggested that, after the PEG-IFNα/RBV therapy, HCV-1b was partially replaced by HCV-1a.

Similarly, the shift in the melting curve of HVR1 cDNA after the therapy and an appreciable change in the T_m_, which differed by over 1 °C, indicated that major changes in the nucleotide sequence of HVR1 cDNA had taken place.

Before the therapy, the sequencing analysis showed two closely related species almost identical with the sequence of the HVR1 typical of HCV-1b. After therapy, three species were detected. Two species showed 59 % homology each to the HVR1 of HCV-1b, whereas the third species of the virus showed 85 % homology to the HVR1 of HCV-1a, indicating that, during therapy, the initial sequence corresponding to HCV-1b was changed to HCV-1a. This suggested that the therapy resulted in a change from a more dangerous 1b to a less perilous 1a subtype of the virus. These results confirmed the previous observations (Berg et al. 2000; Kmieciak et al. 2009) that a higher frequency of mutations in the ISDR was associated with stronger response to therapy. Before therapy, HCV-1a was undetectable, probably due to a very low titer. During therapy, HCV-1b, gradually eliminated by interferon, was partially replaced by HCV-1a, which was more resistant than HCV-1b to the antiviral therapy (Kmieciak et al. 2005).

In our patient, after PEG-IFNα/RBV therapy, among clones containing PKR-BD cDNA, we identified an aa. sequence characteristic of HCV-1a, which is the typical subtype in the northern European population, while in about 40 patients from the same group, only the HCV-1b subtype was detected. This is understandable, since over 90 % of HCV-infected patients in this country are infected by HCV-1b (Simmonds 2004; Kmieciak 2005).

## Conclusions

The novel aspect of our study is the application of the high-resolution melting (HRM) technique for the rapid screening of sequence variations in the regions of hepatitis C virus (HCV) encoding the protein kinase R-binding domain (PKR-BD) region and the hypervariable region 1 (HVR1). This technique has never been used for that purpose. Our results suggest that HRM analysis helps to rapidly detect HCV subspecies in an attempt to evaluate the effectiveness of PEG-IFNα/RBV therapy, and that this technique can be applied for the rapid screening of sequence variations in any viral genome. However, despite its easiness, low costs, and speed, the application of quantitative real-time polymerase chain reaction (qPCR) HRM analysis for routine diagnostics can be limited because of the use of nonspecific dyes.
